# Synthetic *Plasmodium*-Like Hemozoin Activates the Immune Response: A Morphology - Function Study

**DOI:** 10.1371/journal.pone.0006957

**Published:** 2009-09-09

**Authors:** Maritza Jaramillo, Marie-Josée Bellemare, Caroline Martel, Marina Tiemi Shio, Ana Paulina Contreras, Marianne Godbout, Michel Roger, Eric Gaudreault, Jean Gosselin, D. Scott Bohle, Martin Olivier

**Affiliations:** 1 The Research Institute of the McGill University Health Centre, Centre for the Study of Host Resistance, Departments of Medicine, Microbiology and Immunology, McGill University, Montréal, Canada; 2 Department of Chemistry, McGill University, Montréal, Canada; 3 Department of Biochemistry and Goodman Cancer Centre, McGill University, Montréal, Canada; 4 Laboratory of Immunogenetics, Centre de Recherche du Centre Hospitalier de l'Université de Montréal, and the Department of Microbiology and Immunology, Université de Montréal, Montréal, Canada; 5 Laboratory of Viral Immunology, Rheumatology and Immunology Research Center, Centre Hospitalier de l'Université Laval Research Center, Québec, Canada; INSERM U567, Institut Cochin, France

## Abstract

Increasing evidence points to an important role for hemozoin (HZ), the malaria pigment, in the immunopathology related to this infection. However, there is no consensus as to whether HZ exerts its immunostimulatory activity in absence of other parasite or host components. Contamination of native HZ preparations and the lack of a unified protocol to produce crystals that mimic those of *Plasmodium* HZ (*P*HZ) are major technical limitants when performing functional studies with HZ. In fact, the most commonly used methods generate a heterogeneous nanocrystalline material. Thus, it is likely that such aggregates do not resemble to *P*HZ and differ in their inflammatory properties. To address this issue, the present study was designed to establish whether synthetic HZ (sHZ) crystals produced by different methods vary in their morphology and in their ability to activate immune responses. We report a new method of HZ synthesis (the precise aqueous acid-catalyzed method) that yields homogeneous sHZ crystals (*Plasmodium*-like HZ) which are very similar to *P*HZ in their size and physicochemical properties. Importantly, these crystals are devoid of protein and DNA contamination. Of interest, structure-function studies revealed that the size and shape of the synthetic crystals influences their ability to activate inflammatory responses (e.g. nitric oxide, chemokine and cytokine mRNA) *in vitro* and *in vivo*. In summary, our data confirm that sHZ possesses immunostimulatory properties and underline the importance of verifying by electron microscopy both the morphology and homogeneity of the synthetic crystals to ensure that they closely resemble those of the parasite. Periodic quality control experiments and unification of the method of HZ synthesis are key steps to unravel the role of HZ in malaria immunopathology.

## Introduction

*Plasmodium falciparum* causes the most severe form of malaria, an infection that affects 300 to 500 million individuals worldwide and leads to the death of 1.5 to 2.7 million every year [1]. Hyperactivation of the immune response in malaria patients is characterized by elevated levels of various cytokines (IFNγ, TNFα, IL-1, IL-6), chemokines (IL-8, MIP-1α) and reactive nitrogen oxides [2], [3], [4]. Whereas the generation of these proinflammatory molecules favors the reduction of the parasitic load, their exacerbated production participates in the development of malaria immunopathology [2], [5].

Increasing evidence suggests that the parasite metabolite hemozoin (HZ), released in circulation during the intra-erythrocytic cycle, activates the immune response to *Plasmodium* infection. Initially, HZ was only considered as a metabolic waste to detoxify the heme released during hemoglobin digestion by the parasite [6]. Interestingly, HZ accumulation was detected in phagocytic cells and in various organs (*e.g*., liver, spleen and brain) in correlation with disease severity [7], [8], [9], [10]. However, during the past ten years HZ has emerged as a potent immunoactivator both *in vitro* and *in vivo*. Human monocytes and murine macrophages (Mφ) incubated with either *Plasmodium* HZ (*P*HZ) or synthetic HZ (sHZ) produce large amounts of cytokines (IL-1β, TNFα, IL-12), chemokines (MIP-1α, MIP-1β), adhesion molecules (CD11/CD18) and Mφ migration inhibitory factor (MIF) [11], [12], [13], [14], [15], [16], [17], [18]. Moreover, *P*HZ and sHZ amplify IFNγ-mediated nitric oxide (NO) production in murine Mφ [19], [20]. In line with these studies, mouse injection with sHZ rapidly induces the generation of inflammatory mediators including chemokines (MIP-1α/β, MIP-2, MCP-1), cytokines (IL-1β, IL-12) and myeloid-related proteins [21]. Of interest, activation of innate immune responses by HZ was reported to be toll-like receptor 9 (TLR9)-dependent both *in vitro* and *in vivo* [22].

These data point to a role for HZ in the development of various aspects of malaria pathology (*e.g*., fever, anemia, hepatosplenomegaly, cerebral malaria). However, there is a current controversy about the ability of HZ to regulate immune functions. In this regard, Parroche and colleagues [23] claim that HZ is immunologically inert and attribute its previously described inflammatory activity to the stimulation of TLR9 by the parasitic DNA bound to HZ. This discrepancy could be potentially explained by differences in the purification procedures to isolate the native HZ pigment as well as in the methods of preparation of the synthetic HZ crystals. Chemically, HZ is a stable dimer of iron(III)(protoporphyrin[PP]-IX), hematin anhydride also known as β-hematin. It is profoundly insoluble and consists of heme units dimerized through reciprocal iron-carboxylate bonds [24]. The dimers are linked to adjacent ones via hydrogen bonding between the free propionate side-chains. The parasite and synthetic materials are isostructural [25] but there are large differences in the crystal morphologies from different preparations. *P*HZ crystals are remarkably uniform in size and shape, and only certain protocols allow for the isolation of synthetic crystals with these characteristics [26], [27], [28]. Conversely, many techniques yield material that is poorly crystalline and is rather heterogenous and often nanocrystalline [29].

In the present study, we report the generation of new sHZ preparations (rapid crystalline) (rc) HZ that highly resemble the native *P*HZ crystals in both size (0.5–1.0 µm) and physicochemical properties. Importantly, these crystals are devoid of protein and DNA contamination. This latter observation is extended by confocal microscopy which shows that *P*HZ and parasitic DNA do not colocalize *in situ* in the infected red blood cell (iRBC) isolated from *P. chabaudi*-infected mice. Of interest, morphology-function studies reveal that the size and shape of the synthetic crystals (nanocrystalline, slow crystalline and rapid crystalline) influences their ability to activate inflammatory responses, as illustrated by differences in chemokine mRNA induction *in vitro* and *in vivo* and in the amplification of IFNγ-mediated NO production. Altogether, our data demonstrate that sHZ possesses immunostimulatory properties and underline the importance to select an appropriate procedure to obtain highly purified synthetic HZ crystals that closely resemble those of the parasite. Determination of crystallite homogeneity and crystallinity by electron microscopy and X-ray powder diffraction of any preparation are quality control steps which, in conjunction with a suitable HZ synthesis, constitute useful tools to unravel the contribution of HZ in malaria immunopathology.

## Materials and Methods

### Materials

Highly pure crystalline hemin, iron(III)(protoporphyrin-IX)Cl, was purchased from Fluka Chemie (Buchs, Switzerland). TLR2 and TLR4 agonists, respectively lipoteichoic acid (LTA) and lipopolysaccharides (LPS, *Escherichia coli* serotype 0111:B4), were obtained from Sigma-Aldrich (St. Louis, MO). Polyinosine-polycytidylic (poly(I:C)), a synthetic analog of dsRNA used to stimulate TLR3, was also purchased from Sigma-Aldrich. The 2216 sequence CpG-ODN was used as TLR9 ligand (Invivogen, San Diego, CA). IFNγ was provided by Cedarlane Laboratories (Burlington, ON, Canada). Isotopes [α-^32^P]dUTP (3000 Ci/mmol) and [γ-^32^P]dATP (3000 Ci/mmol) were purchased from PerkinElmer (Wellesley, MA).

### Mammalian Cell and Parasite Cultures

TLR4 −/− and MyD88 −/− bone marrow-derived Mφ generated from TLR4- and MyD88-deficient mice and the murine Mφ cell line B10R, isolated from the bone marrow of B10A.Bcgr (B10R) mice [30], were kindly provided by Dr. Danuta Radzioch (McGill University, Montréal, Canada). The human embryonic kidney HEK293 cell line was obtained from ATCC (CRL-1573, Manassas, VA). Cells were cultured in DMEM (Life Technologies, Rockville, MD) supplemented with 10% heat-inactivated fetal bovine serum (FBS) (Wisent, Saint-Bruno, Quebec, Canada) plus 100 µg/ml penicillin, streptomycin and 2 mM L-glutamine at 37°C and 5% CO_2_. In the case of HEK293 cells, gentamicin (30 µg/ml) was also added to the culture media. Promastigotes of the *Leishmania major* A2 strain were maintained in SDM-79 medium at 25°C supplemented with 10% heat inactivated FBS and 5 mg/ml of hemin [31].

### Synthetic Hemozoin (sHZ) Preparations

#### A. Rapid Crystalline HZ (rcHZ): Aqueous Acid-Catalyzed Hematin Anhydride

Hemin (0.8 mmol, 500 mg) was dissolved in degassed NaOH (0.1M, 100 ml) during 30 min with mild stirring. Propionic acid was added drop wise over a 20-min period until a pH of about 4 was achieved. Stirring was stopped and the mixture was heated at 70°C for 18 hours. After cooling the solid was separated and washed as follows: three NaHCO_3_ (0.1M) washes for 3 hours were alternated with MilliQ water. Finally, MeOH and MilliQ water were alternately used to wash 3 times. The sample was then dried in a vacuum oven overnight over phosphorus pentoxide. All hematin anhydride samples were characterized by XRD, IR and FEG_SEM. Yield: 350 mg, 72%. Anal. Calcd. for C_68_H_62_N_8_O_8_Fe_2_: C, 66.35; H, 5.08; and N, 9.10%; Found: C, 66.53; H, 5.26; and N 8.93%.

#### B. Slow Crystalline HZ (scHZ): Anhydrous Base-Annealing Hematin Anhydride

Hemin (0.8 mmol, 500 mg) was transferred in an inert atmosphere box. Sufficient 2,6-lutidine (10 ml) was added to dissolve it completely. The solution was diluted by the addition of 1∶1 methanol: dimethyl sulfoxide (100 ml). The flask was then sealed, wrapped in aluminum foil and taken out of the box. It was allowed to stand undisturbed for anywhere between 2 weeks to 15 months. The flask was then opened and the black mixture was centrifuged at 7000 rpm for 1 hour, and the supernatant decanted. The crystals were then washed once with NaHCO_3_ (0.1M) for 3 hours. The final washes were with alternating MilliQ purified water and methanol for 3 times each. The sample was dried in vacuum oven at 100°C overnight Anal. Calcd. for C_68_H_62_N_8_O_8_Fe_2_: C, 66.35; H, 5.08; and N, 9.10%; Found: C, 65.90; H, 4.90; and N 8.92%.

#### C. Heterogenous nanocrystalline HZ (nHZ): Irregular Aggregates of Hematin Anhydride

Alterations of method A, including different hemin concentration, rapid acid addition, non-stirring during hemin dissolution and/or acid addition, and higher temperature during the annealing stage resulted in amorphous material.

### X-Ray Powder Diffraction (XRD)

Powder diffraction data were collected on a Siemens D5000 X-ray diffractometer using a Cu K_α_ radiation source (*K_α1_* λ = 1.540562 Å). Samples were pulverized and transferred into a 1 mm diameter glass capillary. The latter was aligned with the beam but was not rotated, and held by a polypropylene capillary holder. X-ray powder diffraction was the preferred method to ensure homogeneity of these samples with diagnostic FWHM values being 0.30°, and 0.28° for the hkl = (0,3,1) and (1,3,1) peaks.

### Field Emission Gun Scanning Electron Microscopy (FEG_SEM)

SEM pictures were acquired using a Hitachi S-4700 FEG_SEM. The samples were coated with Au/Pd of about 4 Å in thickness prior to visualization at 2 kV and 10 µA.

### Attenuated Total Reflection-Infrared (ATR-IR) Spectroscopy

Spectra were obtained as the crystals were pressed on a diamond MIRacle ATR cell from PIKE Technology installed on a Perkin-Elmer FTIR BX System and running with Spectrum software. Hematin anhydride is an insoluble black solid with strong characteristic carboxylate bands at 1709, 1663, and 1210 cm^−1^ in the infrared spectrum.

### DNA Extraction and Agarose Gels

DNA from *Leishmania major* promastigotes and B10R Mφ was extracted using DNeasy Blood and Tissue Kit (Qiagen). Purified DNA was incubated with the various HZ crystals (nHZ, scHZ and rcHZ). To visualize the DNA contents, the samples were run in a 1% agarose gel containing 0.5 µg/ml ethidium bromide.

### SDS-PAGE and Silver Staining

Synthetic HZ crystals (100 µg per reaction) were resuspended in DMEM either containing 10% FBS or 5 µg BSA. As negative controls, HZ preparations were diluted in serum-free DMEM. The various samples were incubated at 37°C for 30 min to promote protein binding to HZ. After incubation on ice, the protein-HZ mixtures were centrifuged at 7000 rpm for 15 min at 4°C. Pellets were recovered and washed 3 times by resuspension in 1 ml of endotoxin-free PBS (Life Technologies) and centrifugation (7000 rpm for 15 min at 4°C). Next, samples were prepared for SDS-PAGE followed by silver staining to visualize the proteins bound to the sHZ crystals.

### Mouse Infection and Confocal Imaging of Plasmodium-infected Red blood Cells

The research involving animals in this work was approved by the McGill University Animal Care Committee. Thus, this study was conducted adhering to McGill University's guidelines for animal husbandry. BALB/c mice were injected intraperitonally with *Plasmodium chabaudi* DK-iRBCs, as previously described [32]. Infection was followed over a 15-day period by monitoring the percentage of *Plasmodium*-iRBC in Giemsa-stained blood smears. Blood smears of day 11 post-infection were used to perform confocal microscopy. At that time the percentage of iRBC was 15–20% and several of them contained trophozoites or schizonts. DAPI (Molecular Probes, Burlington, Ontario, Canada) staining was used to visualize parasite nuclear and kinetoplast DNA. Samples were prepared as for fluorescence spectrum on microscope slides with Permafluor and visualized using the 2-photons Titanium: Sapphire laser excitation 750 nm and a band-path emission filter (BP390-465) nm on a Zeiss LSM 510 NLO. Hemozoin co-localization could be visualized by exciting with a 632.5 nm Helium-Neon laser and capturing from 650 to 710 nm.

### Electrophoretic Mobility Shift Assay (EMSA)

Cell stimulation was terminated by the addition of ice-cold PBS, nuclear extracts were prepared according to the microscale protocol and EMSA was performed using 6 µg of nuclear proteins, as described elsewhere [19]. Briefly, nuclear extracts were incubated for 20 minutes at room temperature in 1.0 µl of binding buffer (100 mM HEPES, pH 7.9, 40% glycerol, 10% Ficoll, 250 mM KCl, 10 mM DTT, 5 mM EDTA, 250 mM NaCl), 2 µg of poly(dI-dC) and 10 µg of nuclease-free BSA containing 1.0 ng of [γ-^32^P]dATP radio-labeled dsDNA oligonucleotide. This mixture was incubated for 20 minutes at room temperature and the reaction was stopped using 5 µl of 0.2 M EDTA. DNA-protein complexes were resolved from free-labeled DNA by electrophoresis in native 4% (w/v) polyacrylamide gels containing 50 mM Tris-HCl, pH 8.5, 200 mM glycine, and 1 mM EDTA. The gels were subsequently dried and autoradiographed. The dsDNA oligonucleotide containing a consensus binding site for NF-κB/c-Rel homodimeric and heterodimeric complexes, 5′-AGTTGAGGGGACTTTCCCAGGC-3′ was obtained from Santa Cruz Biotechnology (Santa Cruz, CA). The non-specific probe Oct-2A 5′-GGAGTATCCAGCTCCGTAGCATGCAAATCCTCTGG -3′ was used to confirm the specificity of the DNA/nuclear protein reaction. Cold competitor assays were carried out adding a 100-fold molar excess of homologous unlabeled oligonucleotides of the labeled dsDNA probe.

### Cell transfections and Luciferase assays

HEK293 cells were seeded at 5×10^4^ cells per well in a 24-well plate. Twenty-four hours later, cells were transiently co-transfected with 0.3 µg of selected TLR expressing vectors (pUNO-hTLR-HA) along with 0.1 µg of NF-κB luciferase reporter plasmid (Invivogen, San Diego, CA) using Escort V reagent (Sigma-Aldrich, ON, Canada). Forty-eight hours post-transfection, culture medium was replaced with fresh DMEM containing 10% FBS and stimulated with sHZ crystals (200 µg/ml) or with respective TLR ligands: 10 µg/ml LTA (TLR2), 10 µg/ml poly(I:C) (TLR3), 1 µg/ml LPS (TLR4), 10 µg/ml CpG2216 (TLR9), for 8 hours. Next, cells were lysed in luciferase buffer (1% Triton-X-100, 10% glycerol, 20 mM tris phosphate, pH 7.8) and luciferase activity was measured by luminometry. Relative light units (RLU) were normalized to protein contents. Data were analyzed by one-tailed analysis of variance (ANOVA) followed by Newman-Keuls post-hoc test using PRISM3 software. Differences were considered significant at a *P* of ≤0.05. All data are presented as mean + SEM.

### Nitric Oxide (NO) Generation

Mφ were seeded in 24-well plates (5×10^5^ cells per well). The next day cells were treated for 24 hrs with 100 U/ml IFNγ, 5–50 µg/ml sHZ or a combination of both. NO production was assessed by measuring the accumulation of nitrite in the cell culture medium (Griess reaction), as described elsewhere [19]. Statistically significant differences were determined by ANOVA and the Fisher least significant difference test. Values of *p*<0.05 were deemed statistically significant. All data are presented as mean + SEM.

### In vivo Injection of Synthetic Hemozoin

BALB/c mice were injected into the tail vein with 750 µg sHZ. As a negative control, 100 µl of endotoxin-free PBS were injected intravenously. After 6 h, mice were lethally exposed to CO_2_. Sections of liver tissue were collected and ground in Trizol reagent (Life Technologies) to isolate total RNA according to the manufacturer's protocol. Samples were stored at −80°C for subsequent RNase protection assays and RT-qPCR analysis.

### RNase Protection Assays (RPA)

The mRNA expression studies were performed using a RPA kit (Riboquant, BD Pharmingen), as we described previously [21]. Total RNA was isolated from Mφ or mouse liver tissue, as described above. The multiprobe template was labeled with [α-^32^P]dUTP using T7 RNA polymerase. Then, 3×10^5^ cpm of labeled probe was allowed to hybridize with 10 µg of total RNA for 16 h at 56°C. mRNA probe hybrids were treated with RNase A and phenol-chloroform extracted. Protected hybrids were resolved on a 5% denaturing polyacrylamide sequencing gel and exposed to radiographic film. The Multiprobe template was mCK-5 (BD Pharmingen) for the murine chemokines Lymphotactin, RANTES, Eotaxin, MIP-1α, MIP-1β, MIP-2, IP-10, MCP-1 TCA-3, and included the housekeeping genes mL-32 and GAPDH.

### RT-qPCR Analysis

To monitor the expression of a large panel of inflammatory genes in the liver, total RNA extraction by Trizol was followed by reverse transcriptase (RT) and quantitative PCR (qPCR) analysis using a mouse inflammatory cytokine and receptor RT^2^ Profiler^TM^ PCR array (SuperArray Biosciences, Frederick, MD), as indicated by the manufacturer protocol. Statistically significant differences between groups were determined by the T-Student test. Values of *p*<0.05 were deemed statistically significant.

### Western blot analysis

Following stimulation, bone marrow-derived Mφ from wild type (WT), TLR4−/− or MyD88−/− mice were collected and lysed in cold buffer containing 1×PBS, 1× inhibitor protease cocktail (Roche, Mississauga, Ontario, Canada), 20% Glycerol, 2 mM Na_3_VO_4_ and 1 mM NaF. Cell debris was removed by centrifugation at 13,000 rpm×15 min at 4°C and total protein content was determined using the Bio-Rad Protein assay (Bio-Rad, Mississauga, ON). Lysates were subjected to 10% SDS-PAGE and the separated proteins were transferred onto a PVDF membrane (Bio-Rad). After a one-hour blocking period in TBS-0.1% Tween-20 containing 5% skim milk, the membranes were incubated overnight with one of the following specific antibodies: anti-phospho-JNK, anti-JNK (Cell Signaling, Pickering, Ontario, Canada), anti-phospho-Syk (Upstate Biotechnology, Lake Placid, NY) or anti-Syk (Santa Cruz Biotechnology). Proteins were detected with a secondary goat anti-rabbit horseradish peroxidase-conjugated antibody (Sigma-Aldrich) and subsequent visualization by Western Lightning-ECL (Perkin Elmer).

## Results

### Characterization of Different Preparations of Synthetic Hemozoin

**Figure 1:**
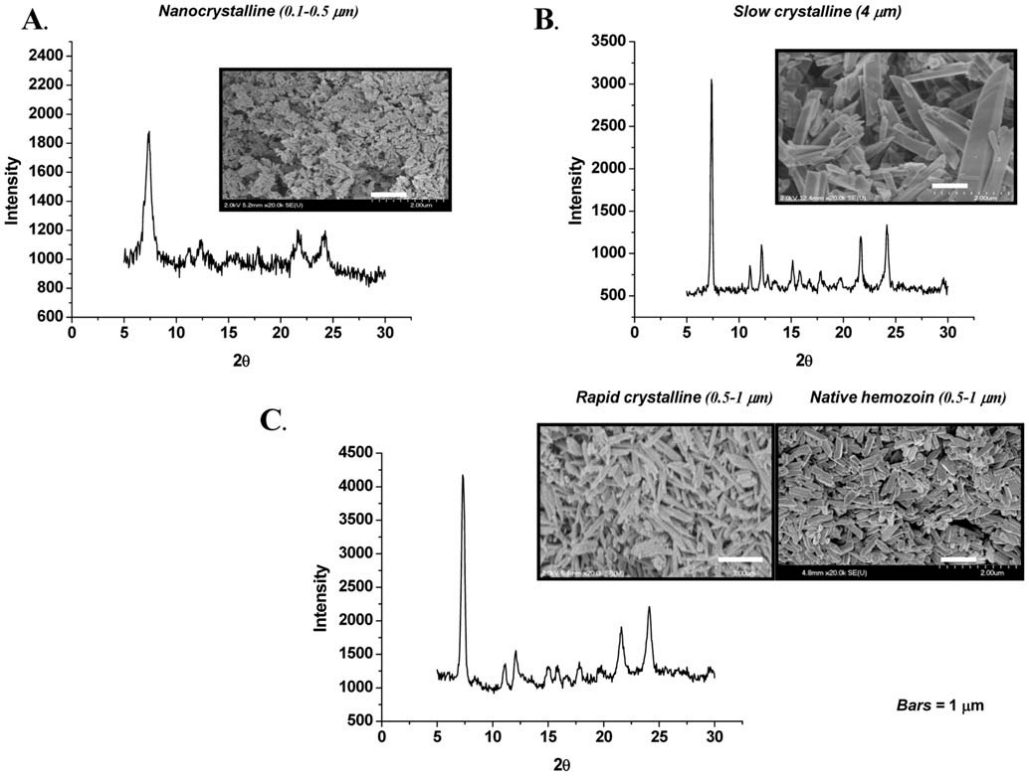
Comparative Analysis of Different Synthetic Hemozoin Preparations. Scanning electron micrographs and X-ray powder diffraction patterns (Cu K*_α1_*) of (A) nanocrystalline HZ (nHZ), (B) slow crystalline HZ (scHZ), (C) rapid crystalline HZ (rcHZ) and *P*HZ. The scale bar in each case is 1 µm.

**Figure 2:**
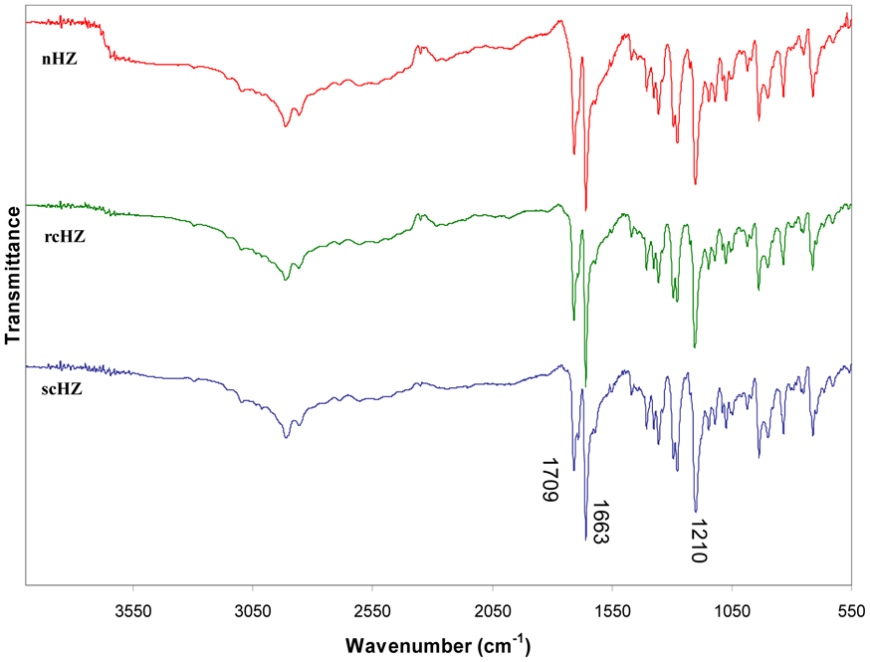
ATR-IR Spectra of the various Hemozoin Crystals. Comparison of the infrared spectra of the sHZ crystals produced by the acid-catalyzed thermal method, nHZ, (red); the aqueous acid-catalyzed method, rcHZ, (*green*); and the anhydrous base-catalyzed method, scHZ, (blue).

Even though HZ has become an important subject of investigation, it is not generally accepted that it has the potential to regulate immune functions in the absence of other parasite- and/or host-derived cofactors, such as DNA. Since most of the native HZ preparations are contaminated with other pasitic components, it would be very helpful to employ a unified protocol that yields *Plasmodium*-like HZ crystals of biomimetic uniform homogeneity and morphology quality. This would allow to validate data obtained from funtional studies performed independently. Therefore, we initially investigated whether sHZ crystals produced by various methods differ physicochemically and morphologically. To this end, three different protocols to prepare sHZ were conducted, as described in the Materials and Methods section. Briefly, the precise aqueous acid-catalyzed protocol and the broader one (heterogeneous nanocrystalline), as well as the anhydrous base-catalyzed method were compared. The resulting samples were assessed for crystal size, morphology and homogeneity by a combination of scanning electron microscopy, X-ray powder diffraction and infrared spectroscopy. As depicted in Figure 1A, the uncontrolled aqueous acid-catalyzed sHZ protocol resulted in a poorly crystalline and heterogeneous nanocrystalline material with an average crystallite size of 0.06–0.14 µm characterized by high surface areas that have a random distribution of surface sites. Broad XRD peaks with FWHM values being 0.78°, and 0.76° for the hkl = (0,3,1) and (1,3,1) peaks confirm the fine size of the crystalline domains. We thus defined this type of preparation as nanocrystalline HZ (nHZ). By contrast, the anhydrous base-catalyzed reaction yielded large crystals that are isomorphous and isostructural with *P*HZ and whose sharp XRD peaks reveal the large crystalline domains (Figure 1B). Since this kind of sHZ was produced only after 12 months of reaction, we named it slow crystalline HZ (scHZ). Of interest, the precise aqueous acid-catalyzed method allowed to generate homogeneous crystals of 0.8–1.0 µm (Figure 1C), which are very similar in size, to those of *Plasmodium* [33], [34], [35]. Because of their resemblance to the parasite HZ and due to the shorter time required to synthesize them, we referred to these crystals as *Plasmodium*-like rapid crystalline HZ (rcHZ). Although the prepared materials differ in crystallinity and morphology, confirmation of the material composition and local structure comes from the ATR-IR (Figure 2). All three spectra showed the characteristic peaks recognized for hematin anhydride which are: the two carboxylate asymmetric stretches at 1709 and 1663 cm^−1^ and the symmetric one at 1210 cm^−1^. Overall, this set of experiments demonstrate that it is possible to obtain crystals (rcHZ) that resemble those found in nature and which can be generated significantly faster than other preparations (scHZ) by performing the aqueous acid-catalyzed method reported here. Noteworthy, our data underscore the importance to avoid procedures that generate nanocrystalline preparations of sHZ rather than larger crystalline forms, and to carry out systematic control experiments to verify the quality of the sHZ crystals to be employed in functional studies.

### Plasmodium Hemozoin and DNA do not Colocalize in Infected Red Blood Cells

**Figure 3:**
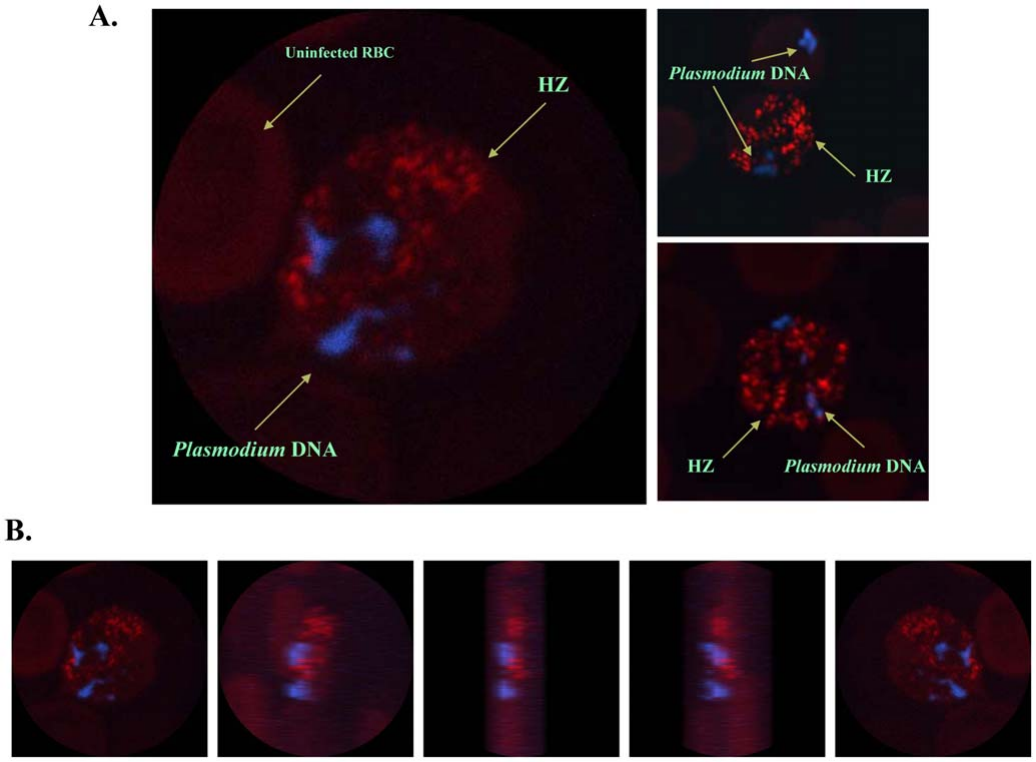
*In situ* Localization of *Plasmodium* Hemozoin and Parasitic DNA. Confocal pictures of RBC from *Plasmodium chabaudi* DK-infected mice. (A) Selected images of schizonts and late trophozoites stages of iRBC. (B) Selected views from a z-stack performed on a late trophozoite iRBC. DAPI staining was used to visualize parasitic DNA (blue). No staining was required to localize HZ since it autofluoresces (red). Images were taken from a 63X objective of a Zeiss LSM-150 microscope. Immunofluorescence analysis was performed three times and the most representative results are displayed.

**Figure 4:**
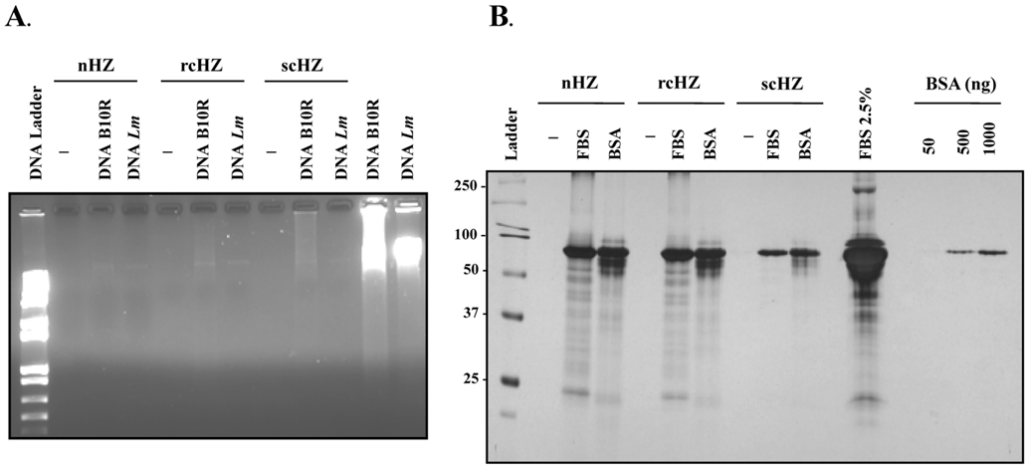
Interaction of Synthetic Hemozoin Crystals with DNA or Proteins. (A) 100 µg of nHZ, rcHZ or scHZ were left untreated or were incubated with 1 µg of DNA extracted from B10R Mφ or from promastigote cultures of *Leishmania major*. After washes, recovered pellets were resuspended in sample loading buffer and run in a 1% agarose gel containing 0.5 µg/ml ethidium bromide. As positive controls for DNA contents, 1 µg of Mφ or parasite DNA were loaded. (B) SDS-PAGE and silver staining of 100 µg of sHZ crystals (nHZ, rcHZ and scHZ) incubated in absence or in presence of DMEM containing either 10% FBS or 5 µg of BSA. As positive controls for protein contents, 2.5% FBS or increasing concentrations of BSA (50, 500 and 1000 ng/ml) were loaded.

It has been recently claimed that the immune activity attributed to HZ is indirect and rather due to its attachment to parasitic DNA, which in turn activates TLR9-mediated immune responses [23]. In an attempt to address this issue, we performed confocal analysis of iRBC from mice infected with *Plasmodium chabaudi* DK during 11 days. At this time, several iRBC contained trophozoites and schizonts, which are the intraerythrocytic parasite stages with the highest HZ contents. As shown in Figures 3A and 3B, parasitic DNA (blue) and *P*HZ, which fluoresces spontaneously (red), do not colocalize *in situ* in the iRBC. Of interest, we found that even after merozoite release from the iRBC (Figure 3A, *right upper panel*) parasitic DNA is never in contact with *P*HZ. This *in situ* data prompted us to investigate whether sHZ crystals interact with cellular DNA or proteins *in vitro*. To this end, the various sHZ crystals (nHZ, scHZ and rcHZ) were incubated with DNA purified from B10R Mφ or *Leishmania major* parasites. As summarized in Figure 4A, this experiment revealed that the sHZ preparations interact very weakly with either parasitic or mammalian DNA, as evidenced by the much higher amount of DNA encountered in samples that were not incubated with the sHZ crystals. In addition to DNA, it has been proposed that cellular proteins from both host and parasite origin adhere to the crystalline core of HZ [36], [37], [38], [39]. Therefore, we next carried out a similar experiment to the above-described, but this time the sHZ preparations were incubated with DMEM containing either 10% FBS or 5 µg of BSA. SDS-PAGE followed by silver staining revealed that BSA as well as numerous proteins present in serum bind to all the sHZ crystals tested (Figure 4B). In summary, these data provide evidence that whereas proteins adhere to HZ, parasitic and mammalian DNA only slightly interact with sHZ *in vitro*, and parasitic DNA does not colocalize with *P*HZ *in situ*. Therefore, we predict that it is very unlikely that the immune activity attributed to HZ is caused by its ability to bind parasitic DNA. However, host and/or parasitic proteins present in the milieu where HZ is released, may bind to these crystals and could eventually alter their immunostimulatory capacity. Further investigation will shed light on this matter.

### Synthetic Hemozoin induces NF-κB Activity in a TLR-Independent Manner

**Figure 5:**
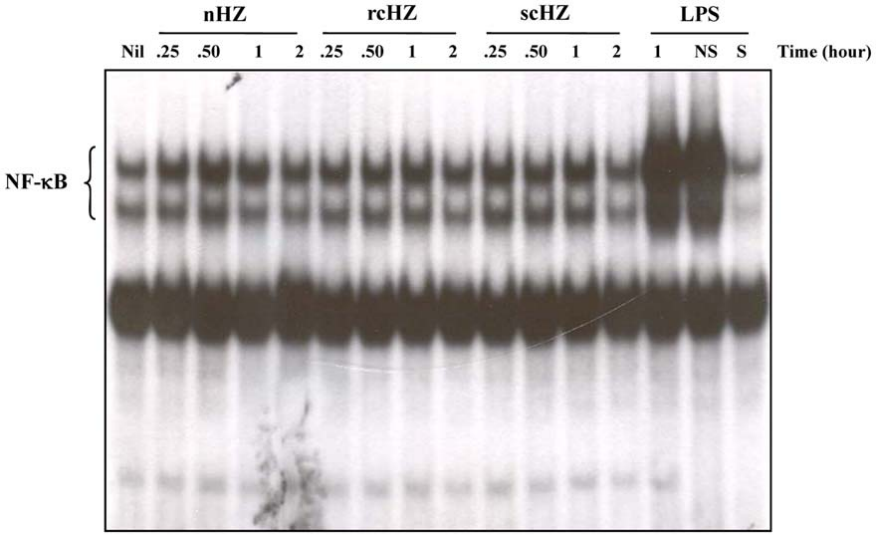
Synthetic Hemozoin Leads to NF-κB Nuclear Translocation Regardless of the Preparation Method. B10R Mφ were either left untreated or stimulated with (50, 100 µg/ml) of nHZ, rcHZ or scHZ for different time periods (0.25, 0.5, 1, 2 hrs). Nuclear proteins were extracted, incubated with a [γ-^32^P]-labeled NF-κB probe and subjected to EMSA. As a positive control for NF-κB induction, Mφ were treated for 1 h with 100 ng/ml of LPS. Binding specificity was tested by adding to nuclear extracts from LPS-stimulated cells a 100-fold molar excess of either a cold NF-κB consensus oligonucleotide (S), or a nonspecific Oct-2A probe (NS). These results are representative of one of three independent experiments.

The above-described data demonstrated that our sHZ preparations are free of contaminants and that depending on the method of synthesis such crystals exhibit profound morphological and physicochemical differences (nHZ versus scHZ and rcHZ). We were next interested to establish whether variations in size and shape of the sHZ crystals had an impact in their ability to trigger intracellular signals. NF-κB is a transcription factor induced in numerous infections in which it promotes the expression of multiple inflammatory mediators [40]. We previously showed that sHZ-mediated nuclear translocation of NF-κB was important for chemokine induction and NO synthesis in the Mφ [18]. Therefore, we monitored changes in Mφ NF-κB activity. As depicted in Figure 5, when B10R Mφ were treated with 50–100 µg/ml of any of our sHZ preparations, a rapid nuclear accumulation of NF-κB was detected (within 15–30 min). However, the intensity and kinetics of induction appeared to vary. In response to nHZ, NF-κB nuclear translocation was stronger, faster and transient. In contrast, scHZ and rcHZ stimulation resulted in a more moderate and sustained NF-κB DNA binding activity.

**Figure 6:**
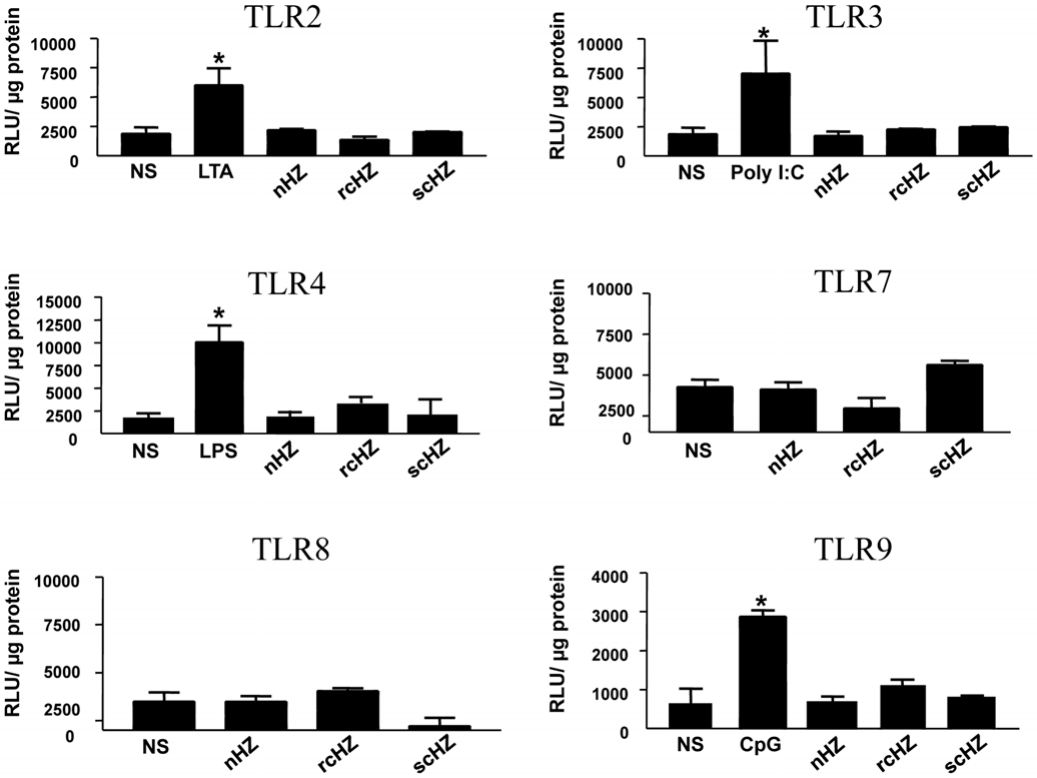
Induction of NF-κB Activity by Synthetic Hemozoin is TLR-Independent. HEK293 (5×10^4^ cells/well) were co-transfected with 0.3 µg of selected TLR expressing vectors (pUNO-hTLR-HA) along with 0.1 µg of a NF-κB luciferase reporter plasmid. Forty-eight hours later, cells were stimulated with nHZ, rcHZ or scHZ (200 µg/ml) for 8 hrs. As positive controls for TLR activation, cells were treated for 8 hrs with the respective TLR ligands; 10 µg/ml LTA (TLR2), 10 µg/ml poly(I:C) (TLR3), 1 µg/ml LPS (TLR4), 10 µg/ml CpG2216 (TLR9). Cells were lysed and luciferase activity was measured by luminometry. Relative light units (RLU) were normalized to protein contents. Experiments were performed in triplicate (mean + SEM, n = 3). *, *p*<0.05, as compared to non-stimulated (NS) cells.

Coban and colleagues [22] reported that the induction of innate immune responses by HZ is TLR9-dependent. Since such immune-responsive genes (e.g., TNFα, MCP-1) contain functional DNA binding sites for NF-κB [41], [42], we set out to elucidate whether TLR9 and/or other TLRs were required for sHZ-mediated NF-κB activity. To accomplish this, HEK293 cells were transiently transfected with pUNO-hTLR-HA expression vectors (TLR2, 3, 4, 7, 8, 9) along with a NF-κB luciferase reporter plasmid. Forty-eight hours post-transfection, cells were stimulated with 200 µg/ml of nHZ, scHZ or rcHZ for 8 h. As controls for TLR activation, cells were treated with their respective TLR ligands. As summarized in Figure 6, while transfected TLRs were found functional when stimulated with their specific ligand, none of the sHZ crystals elicited this type of response. Thus, we conclude that even though all sHZ preparations tested in this study increase nuclear translocation and DNA binding of NF-κB, this event is TLR-independent.

In addition to NF-κB, we explored whether the activation of other Mφ intracellular messengers in response to sHZ is also TLR-independent. We found that sHZ leads to the phosphorylation of Spleen Tyrosine kinase (Syk) not only in wild type (WT) bone marrow-derived Mφ (BMM) but also in TLR4 −/− and MyD88 −/− BMM. By contrast, LPS-mediated Syk phosphorylation was not detected in any of these cells (Figure S1, upper panel). This observation provides evidence that HZ and LPS interact with different Mφ receptors and trigger distinct signaling events. Further supporting this notion, LPS-inducible phosphorylation of JNK was clearly observed in WT BMM and was almost completely abolished in TLR4 −/− and MyD88 −/− BMM; however, sHZ failed to activate JNK in all the cell lines tested (Figure S1, lower panel). These data indicate that HZ activates the Mφ through TLR-independent signals and demonstrate that our sHZ preparations are free of LPS contamination.

### Interferon-γ-Inducible Nitric Oxide Generation is Enhanced by Synthetic Hemozoin

**Figure 7:**
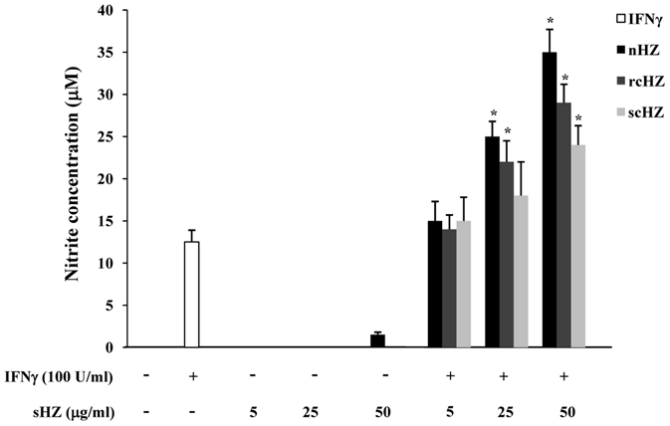
Synthetic Hemozoin Amplifies Interferon-γ-Mediated Nitric Oxide Production. B10R Mφ were stimulated with 100 U/ml IFNγ and/or with increasing concentrations (5, 25, 50 µg/ml) of nHZ, rcHZ or scHZ for 24 h. Then, supernatants were collected and submitted to the colorimetric Griess reaction to evaluate nitrite production. IFNγ (open bars); nHZ ± IFNγ (solid bars); rcHZ ± IFNγ (dark gray bars); scHZ ± IFNγ (light gray bars). Results are representative of one of three independent experiments performed in triplicate (mean + SEM, n = 3). *, *p*<0.05, sHZ + IFNγ versus IFNγ.

NO overproduction appears to contribute to the immunopathology related to malaria infection [43], [44]. Of interest, we and others have reported that *P*HZ and sHZ exert a potent synergistic effect on IFNγ-inducible NO generation [19], [20], [45]. In view of these results, we assessed the capacity of the different sHZ crystals, nHZ, rcHZ and scHZ, to modulate the synthesis of this important inflammatory mediator. To this end, B10R Mφ were stimulated with 100 U/ml IFNγ and/or 5, 25, 50 µg/ml of nHZ, rcHZ or scHZ. After 24 h, cell culture supernatants were collected and nitrite production was monitored. Whereas none of the sHZ preparations augmented NO levels on their own, all of them significantly enhanced IFNγ-mediated NO production in a concentration-dependent fashion (Figure 7). It should be noted that at the highest dose (50 µg/ml) nHZ exerted the greatest up-regulatory effect, reaching up to a 3-fold increase over cells treated with IFNγ alone, while the same amounts of scHZ and rcHZ resulted in a maximum enhancement of ∼2-fold. These results revealed that even though all the sHZ preparations tested are able to positively regulate IFNγ-dependent Mφ functions, nHZ appears to be the most potent of all.

### Synthetic Hemozoin Increases mRNA Levels of Various Pro-inflammatory Molecules *in vitro* and *in vivo*

**Figure 8:**
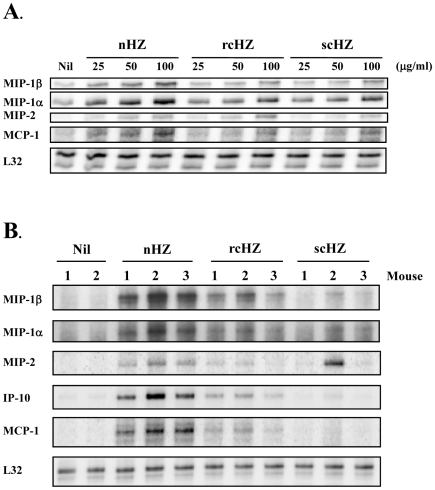
Up-Regulation of Macrophage and Liver Chemokine Transcripts in Response to Synthetic Hemozoin. (A) Cells were treated with increasing concentrations (25, 50, 100 µg/ml) of nHZ, rcHZ or scHZ for 2 h. After total RNA extraction, chemokine mRNA expression was monitored using a mCK5 multi-probe RPA system. Results are representative of one of three independent experiments. (B) BALB/c mice were injected in the tail vein with either PBS or 750 µg of nHZ, rcHZ or scHZ. After a 6-h treatment, mice were lethally exposed to CO_2_ and their livers were dissected and ground. Total RNA extraction was followed by RPA analyses to examine chemokine mRNA levels in liver. Data presented correspond to 2 or 3 animals representative of each experimental group.

Increasing evidence indicates that HZ induces chemokine gene expression *in vitro* and *in vivo*. However, the majority of these studies employed amorphous preparations of sHZ. To test whether more crystalline forms of sHZ (rcHZ and scHZ) also have the ability to augment mRNA chemokine levels, we stimulated B10R Mφ for 2 h with 25, 50 or 100 µg/ml of the different sHZ crystals. As shown in figure 8A, we detected a significant and concentration-dependent increase in MIP-1β, MIP-1α, MIP-2 and MCP-1 transcripts. In concert with our results about NO production, the most powerful chemokine activator was nHZ; however, in a lesser extent, both rcHZ and scHZ also displayed this inflammatory property. Next, we assessed the validity of our *in vitro* data in a murine experimental model. In order to mimic the release of HZ into the bloodstream, 750 µg of nHZ, rcHZ or scHZ were injected into the tail vein of BALB/c mice. HZ effects were monitored in the liver since its accumulation in this organ has been associated with infection chronicity and cumulative parasite burden [10], [46]. Six hours post-treatment, a slight but detectable increase of various liver chemokine transcripts (MIP-1β, MIP-1α and MIP-2) occurred when scHZ was injected. A greater induction of these messengers, in addition to those of IP-10 and MCP-1, was observed in liver samples from rcHZ-treated mice. In support of our *in vitro* data, maximal chemokine mRNA accumulation was found upon nHZ injection (Figure 8B). These results were further extended by performing RT-qPCR analysis of the same liver RNA samples. As summarized in Table 1, this technique allowed us to identify a group of inflammatory genes (*e.g*., MCP-1, MIP-1β, MIP-1α, MCP-3, MCP-5, mouse KC, IP-10, IL-1β, TNFα) that are significantly up-regulated in response to the various sHZ crystals.

**Table 1:** Synthetic Hemozoin-mediated Inflammatory Gene Expression in Liver.

Symbol	Systematic Name (Other names)	Fold Increase		
		nHZ	rcHZ	scHZ
CCL12	Chemokine ligand 12 (MCP-5)	2.6	4.15*	1.70
CCL2	Chemokine ligand 2 (MCP-1, JE)	8.01*	12.6*	3.89
CCL3	Chemokine ligand 3 (MIP-1α)	1.97	3.53*	3.83*
CCL4	Chemokine ligand 4 (MIP-1β)	3.07*	3.50*	4.42*
CCL7	Chemokine ligand 7 (MCP-3, MARC)	4.91*	5.82*	2.71*
CCR2	Chemokine receptor 2	2.48*	1.72	1.18
CXCL1	Chemokine ligand 1 (mouse KC)	24.8*	2.43	1.71
CXCL10	Chemokine ligand 10 (IP-10, CRG-2)	3.78	4.62*	1.71
CXCL13	Chemokine ligand 13 (BLC)	2.44	3.33*	1.59
CXCL4	Chemokine ligand 4 (PF-4)	1.17	1.59*	1.12
CXCL9	Chemokine ligand 9 (Mig, CRG-10)	1.59	3.04*	1.29
IL-1β	Interleukin 1β	3.63	2.70*	1.33
IL-2rg	Interleukin 2 receptor γ chain	1.56	2.16*	1.11
LTB	Lymphotoxin B	1.88*	1.19	1.86
TNF	Tumor necrosis factor	3.23	2.99*	1.17
CD40L	CD40 ligand	1.25	2.20*	1.13

Mouse treatment with the various preparations of sHZ was conducted as described in Figure 8B. After RNA extraction, samples were submitted to RT and qPCR analysis using a RT^2^ Profiler^TM^ PCR array to monitor the expression of multiple inflammatory genes in the liver. Fold increase values were calculated as the average value of three replicas (sHZ sample/untreated sample ratio) and were normalized to GAPDH. *, *p*<0.05, nHZ, rcHZ or scHZ versus untreated cells.

## Discussion

In malaria patients, the number of human leukocytes containing HZ is associated with disease severity [7]. This correlation might be considered simply as a consequence of high parasite burden. However, increasing evidence indicates that HZ interacts with the immune cells, affects their functions and thereby participates in the immunopathology of the infection. In this regard numerous studies using either mouse models or human and murine monocytic cells reported the ability of HZ to induce of a variety of immune-responsive genes. Despite these observations, there is currently no consensus regarding the inflammatory nature of HZ. One of the technical constrains when performing functional studies with HZ is the lack of a unified protocol that yields biomimetic *Plasmodium*-like HZ crystals of uniform homogeneity and morphology. In fact, most of the commonly used synthetic methods for HZ lack precision and generate heterogeneous nanocrystalline aggregates whose physicochemical properties and morphology were not determined [28], [29]. Thus, it is likely that the different methods employed until now result in various types of HZ crystals which potentially diverge in their immunostimulatory properties.

In order to clarify this issue, the present study was designed to generate sHZ crystals using different protocols, to compare them with the native pigment and to establish whether they can activate the immune response. We report a new method of HZ synthesis (the precise aqueous acid-catalyzed method) that yields homogeneous sHZ crystals which are very similar to *P*HZ in their size and physicochemical properties. Because these sHZ crystals were produced relatively faster than others and since they highly resemble those of the parasite, we named them *Plasmodium*-like rapid crystalline HZ (rcHZ). In contrast to these preparations, an uncontrolled variation of the aqueous acid-catalyzed protocol resulted in poorly crystalline aggregates of variable size and much smaller than the *P*HZ; therefore, we identified them as nanocrystalline HZ (nHZ). When the anhydrous base-catalyzed method was employed, we obtained isostructural crystals but four times larger than those generated by the parasite. Because of the amount of time required to prepare them, these crystals were named slow crystalline HZ (scHZ). Despite the structural differences among the various preparations, their IR spectra revealed that all of them are hematin anhydride. However, as evidenced by the electron micrographs and the X-ray powder diffraction patterns, only two of them (rcHZ and scHZ) closely resemble the parasite pigment in terms of crystallinity. These observations are in line with those of Bohle and colleagues [24], [27], who designed the anhydrous base-catalyzed method, which allows the generation of sHZ crystals structurally identical to those of *Plasmodium falciparum*. The disadvantages of this method are the much bigger size of the crystals when compared to the *P*HZ ones, the necessity to work under inert atmosphere and the long period of time required to produce them. However, both problems can now be overcome by adopting the new method of HZ synthesis described herein (the precise aqueous acid-catalyzed method).

Our data revealed that the three different sHZ crystals possess inflammatory activity. This was illustrated by their ability to increase chemokine and cytokine transcript levels in Mφ and in the liver of sHZ-injected mice. Consistent with this, we and others have shown chemokine and cytokine induction in Mφ by both *P*HZ and sHZ [11], [12], [18] indicating that the synthetic crystals behave similarly to the native ones. Further support for this proposal is the observation that human PBMCs treated with *P*HZ or sHZ have increased expression of various β-chemokines (*e.g*., MIP-1α, MIP-1β) whose levels also augment in children with severe malaria [47]. In addition to chemokine and cytokine up-regulation, we found that the various crystals enhanced IFNγ-mediated NO synthesis. In concert with these results, we previously described the same effect in either *P*HZ- or sHZ-fed murine Mφ [19]. Importantly, in human PBMCs *P*HZ significantly increased the levels of NOS2 mRNA and NO production in response to an IFNγ and LPS co-treatment. The biological relevance of this observation was strengthened by the finding that NOS activity augments in PBMCs isolated from children suffering of severe malaria [45]. This report is in sharp contrast with a more recent work in human monocytes showing that *P*HZ or sHZ treatment in combination with IFNγ or LPS did not result in enhanced iNOS expression and NO levels whereas both events were detectable in a murine Mφ cell line [20]. These inconsistencies probably stem from the fact that while the first study was performed in a mixed population of PBMCs which is likely to contain NO-producing cells, the latter report used purified preparations of human monocytes, which synthesize much lower amounts of iNOS and NO [48], [49]. A more detailed study is required to establish the main cellular source of NO in malaria patients and to determine whether HZ is at least in part responsible for such event.

We and others have previously investigated the intracellular signals involved in the activation of Mφ immune-responsive genes by HZ [18], [19], [22]. Among these intracellular messengers, we identified the transcription factor NF-κB, which participates in HZ-dependent Mφ chemokine and NO up-regulation [18], [19]. As expected, we detected NF-κB nuclear translocation in response to our three sHZ preparations. Moreover, transcriptional assays revealed that this event is TLR-independent. Upon first consideration, this would seem inconsistent with previous data indicating that TLR engagement by HZ is necessary for induction of proinflammatory genes [22]. This discrepancy might be related to differences in the experimental systems used in both studies. First, we explored the requirement of TLRs in NF-κB induction while the above-mentioned report investigated TLR involvement in the expression of various inflammatory molecules (*e.g*., TNFα, MCP-1, IL-12). Second, whereas we employed HEK cells to assess the contribution of TLRs to NF-κB activity, Coban and colleagues [22] monitored the role of these receptors in sHZ-inducible DC and spleen cell functions. Thus, it is conceivable that HZ interacts with different receptors in diverse cell types. In this regard, we recently reported that HZ leads to IL-1β production in BMM through the activation of the NOD-like receptor containing pyrin domain 3 (NLRP3) inflammasome [50]. This event required the phosphorylation of the upstream kinase Syk, which was independent of the TLR adaptor molecule MyD88. Confirming and extending these data, we found that sHZ-mediated Syk phosphorylation not only occurs in absence of MyD88 but is also independent of TLR4. Whereas activation of Syk was detected in sHZ-stimulated cells, LPS, a TLR4 ligand, did not exert this effect. Conversely, sHZ failed to phosphorylate JNK while LPS activated this kinase in a TLR4- and MyD88-dependent manner. Thus, we conclude that sHZ and LPS appear to engage different Mφ receptors and thereby trigger distinct intracellular signals. Importantly, these experiments provide evidence that the sHZ preparations employed in the current study are not contaminated with LPS, as clearly observed when the phosphorylation status of Syk and JNK in response to both stimuli was examined.

The extent to which our various sHZ crystals triggered inflammatory responses *in vitro* and *in vivo* inversely correlated with their size and degree of crystallinity. As described in the Results section, nHZ aggregates were highly proinflammatory whereas scHZ, the largest of all crystals, were much less immunostimulatory. We hypothesize that because of the smaller size of the nHZ aggregates there is greater surface/area ratio of the inductive motif interacting with a single Mφ, thereby triggering more strongly its intracellular signals. This is further suggested by the fact that the biggest crystals, scHZ, generate the weakest up-regulating effect in Mφ functions. A more detailed analysis is required in order to explain these observations. Importantly, the possibility that the inflammatory activity of our sHZ crystals was due to contamination with either DNA or protein was ruled out by monitoring their presence in our various sHZ preparations by ethidium bromide-stained agarose gels and silver staining, respectively. Furthermore, elemental analysis of the various sHZ preparations clearly indicated absence of biomolecules on the surface of the crystals. Altogether, these data confirm that sHZ exerts immunostimulatory effects. However, this does not eliminate the possibility that following HZ release in circulation proteins from both host and parasitic origin enter in contact with the pigment, attach to it and modulate its immune activity. In fact, our data showing that serum proteins as well as purified BSA can bind to sHZ imply that this is likely to occur during the erythrocytic life cycle of the parasite. Future studies employing serum from malaria patients as well as from *Plasmodium*-infected mice will contribute to define the role of HZ-attached proteins in malaria pathogenesis.

In sharp contrast with our findings as well as those of other groups [11], [12], [18], [21], [22], Parroche and colleagues [23] reported that HZ lacks immunostimulatory activity. The authors claim that the contribution of HZ to the activation of the innate immune response lies on its ability to bind parasitic DNA and to target it to the endosome where it can trigger TLR9 activation. Even though it cannot be excluded that *Plasmodium* DNA can enter in contact with HZ during infection, our confocal data clearly show that *P*HZ does not colocalize *in situ* with parasitic DNA inside the iRBC and neither does so after merozoite release into the bloodstream following schizont rupture. Moreover, we observed that parasitic and mammalian DNA weakly interact with the sHZ crystals. Thus, it is conceivable that during the procedure employed by Parroche *et al.*, to isolate native HZ form the parasite, merozoite DNA contamination occurred following iRBC lysis. In addition, since the authors did not provide any information about the structure and physicochemical properties of their native and synthetic preparations, it is possible that the integrity of their crystals was affected by the purification and/or synthesis procedures. This is further suggested by the data presented by Coban and colleagues [22], who performed a very similar study to that of Parroche *et al.*, but in contrast to them they found that both *P*HZ and sHZ were immunologically active. Their opposite results cannot be attributed to cell type specificity since both groups monitored the effects of HZ in DCs. Importantly it cannot be argued that in the Coban study the sHZ preparations were contaminated with DNA since they carried out multiple controls including ethidium bromide-stained agarose gels showing that their sHZ crystals were DNA-free. Significantly, following either DNase treatment or heat-inactivation of their sHZ preparations, these crystals were still able to activate DC functions, as reflected by an increase in IL-12 and TNFα production. Even though functional studies in DCs might be informative, it is important to take into account that the main cells to interact with HZ in the host are monocytes in circulation as well as tissue Mφ [7], [9], [12]. Thus, investigation of the effects of HZ in Mφ functions is more biologically relevant in the context of malaria infection.

In conclusion, our data, along with the above-described evidence, confirm that sHZ possesses immunostimulatory properties and underscore the importance to produce *Plasmodium*-like HZ crystals of high quality. The purpose of evaluating the impact of HZ in the immune response is to assess the contribution of this parasite metabolite in the progression of malaria infection. Thus, we consider of paramount importance to select a unified method that generates synthetic HZ crystals which actually mimic the native ones and to test the HZ preparations with electron microscopy and X-ray powder diffraction to ensure their homogeneity. This is particularly important before using them in functional studies. Only by adopting this practice, the data obtained independently can be validated and will acquire biological relevance in the context of malaria immunopathology, which deserves further investigation at the molecular and cellular levels.

## Supporting Information

Figure S1 TLR4 and MyD88 are not Involved in Synthetic Hemozoin-induced Signaling. Bone marrow-derived Mφ from WT, TLR4 −/− or MyD88 −/− mice (0.5×10^6^ cells/0.5 ml) were seeded in 12-well plates. After 2 hours of adherence, cells were stimulated with sHZ (200 µg/ml) or LPS (100 ng/ml) for 20 min. Total proteins were extracted and subjected to western blot analysis with anti-phospho-JNK, anti-JNK, anti-phospho-Syk or anti-Syk antibodies, as described in the Methods section. Data are representative of three independent experiments.
(0.27 MB TIF)
