# Correction: Synthetic *Plasmodium*-Like Hemozoin Activates the Immune Response: A Morphology - Function Study

**DOI:** 10.1371/annotation/06938481-6c6e-4845-af5c-8d9c8131c4b7

**Published:** 2009-10-07

**Authors:** Maritza Jaramillo, Marie-Josée Bellemare, Caroline Martel, Marina Tiemi Shio, Ana Paulina Contreras, Marianne Godbout, Michel Roger, Eric Gaudreault, Jean Gosselin, D. Scott Bohle, Martin Olivier

The affiliation for the fourth author was incorrect. Marina Tiemi Shio is not affiliated with #3 but with #1 The Research Institute of the McGill University Health Centre, Centre for the Study of Host Resistance, Departments of Medicine, Microbiology and Immunology, McGill University, Montréal, Canada. 

