# Supplementary figures and images for: Synthetic Plasmodium-Like Hemozoin Activates the Immune Response: A Morphology - Function Study
Source: PLoS One. 2009 Sep 9;4(9):e6957. doi: 10.1371/journal.pone.0006957 (PMC2734055; doi:10.1371/journal.pone.0006957)

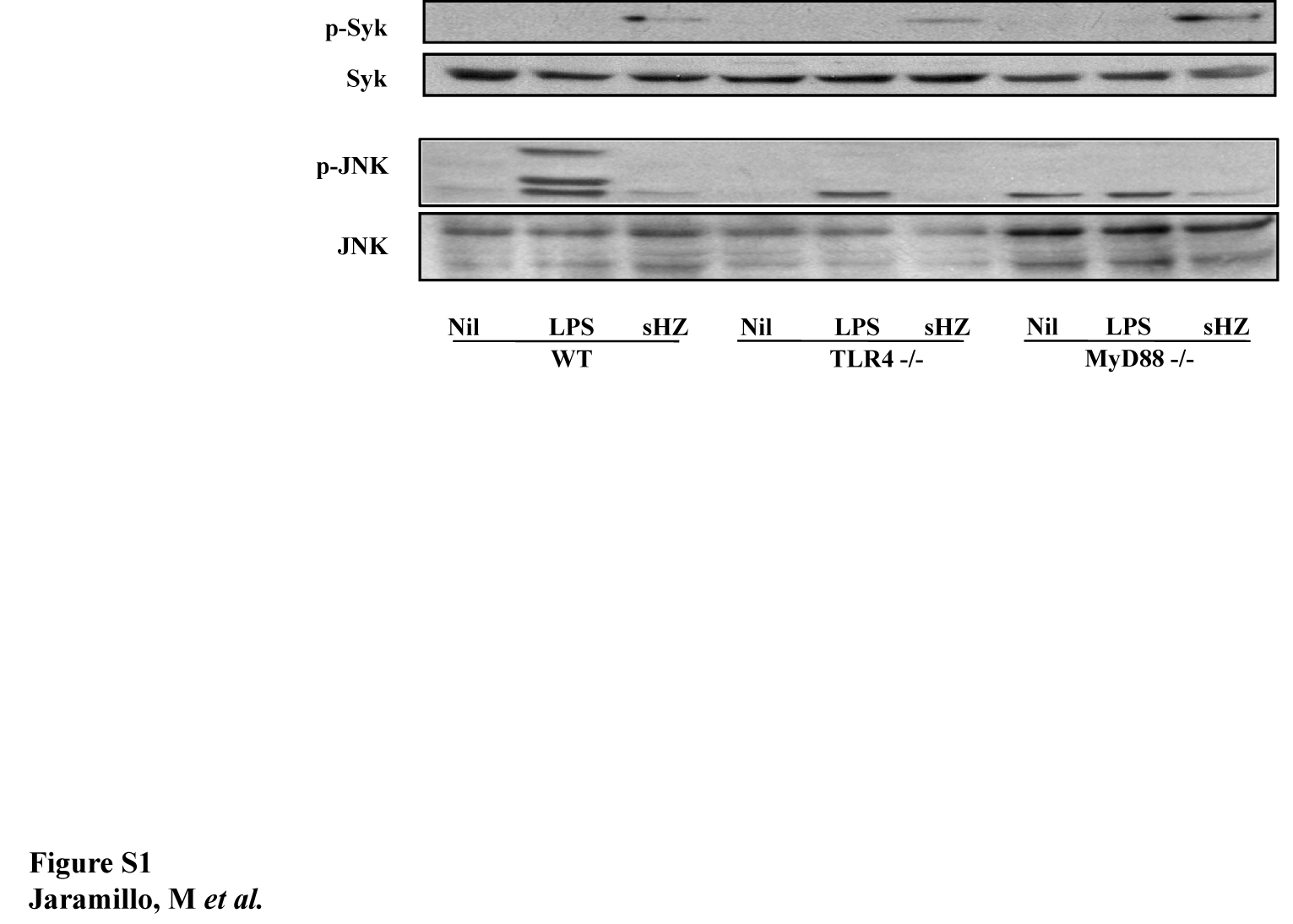

Supplement: Figure S1 — TLR4 and MyD88 are not Involved in Synthetic Hemozoin-induced Signaling. Bone marrow-derived Mφ from WT, TLR4 −/− or MyD88 −/− mice (0.5×10^6^ cells/0.5 ml) were seeded in 12-well plates. After 2 hours of adherence, cells were stimulated with sHZ (200 µg/ml) or LPS (100 ng/ml) for 20 min. Total proteins were extracted and subjected to western blot analysis with anti-phospho-JNK, anti-JNK, anti-phospho-Syk or anti-Syk antibodies, as described in the Methods section. Data are representative of three independent experiments. (0.27 MB TIF) [file pone.0006957.s001.tif]
